# A polymer acceptor with an optimal LUMO energy level for all-polymer solar cells†
†Electronic supplementary information (ESI) available: Experimental details, thermal property, theoretical calculations, as well as all-PSC device fabrications and characterizations. See DOI: 10.1039/c6sc01756h


**DOI:** 10.1039/c6sc01756h

**Published:** 2016-06-14

**Authors:** Zicheng Ding, Xiaojing Long, Chuandong Dou, Jun Liu, Lixiang Wang

**Affiliations:** a State Key Laboratory of Polymer Physics and Chemistry , Changchun Institute of Applied Chemistry , Chinese Academy of Sciences , Changchun 130022 , People's Republic of China . Email: liujun@ciac.ac.cn ; Email: chuandong.dou@ciac.ac.cn; b University of Chinese Academy of Sciences , Beijing 100864 , People's Republic of China

## Abstract

A key parameter for polymer electron acceptors is the lowest unoccupied molecular orbital (LUMO) energy level (*E*_LUMO_). For state-of-the-art polymer electron acceptors based on the naphthalene diimide (NDI) unit, their *E*_LUMO_ are low-lying and cannot be tuned, leading to a low open-circuit voltage (*V*_oc_) of the resulting all-polymer solar cells (all-PSCs). We report that polymer electron acceptors based on the double B←N bridged bipyridine (BNBP) unit exhibit tunable *E*_LUMO_ because of their delocalized LUMOs over polymer backbones. The *E*_LUMO_ of the copolymer of the BNBP unit and selenophene unit (**P-BNBP-Se**) is lower by 0.16 eV than that of the copolymer of the BNBP unit and thiophene unit (**P-BNBP-T**). As a result, the energy levels of **P-BNBP-Se** match well with the widely-used polymer donor, poly[(ethylhexyl-thiophenyl)-benzodithiophene-(ethylhexyl)-thienothiophene] (**PTB7-Th**). The electron mobility of **P-BNBP-Se** (*μ*_e_ = 2.07 × 10^–4^ cm^2^ V^–1^ s^–1^) is also higher than that of **P-BNBP-T** (*μ*_e_ = 7.16 × 10^–5^ cm^2^ V^–1^ s^–1^). While the all-PSC device based on the **PTB7-Th**:**P-BNBP-T** blend shows a moderate power conversion efficiency (PCE) of 2.27%, the corresponding device with **P-BNBP-Se** as the acceptor exhibits a PCE as high as 4.26%. Moreover, owing to the suitable *E*_LUMO_ of **P-BNBP-Se**, the all-PSC device of **P-BNBP-Se** shows a *V*_oc_ up to 1.03 V, which is higher by 0.22 V than that with the conventional NDI-based polymer acceptor. These results indicate that BNBP-based polymers can give all-PSCs with high PCEs, remarkably high *V*_oc_ values and small photon energy losses.

## Abstract 2

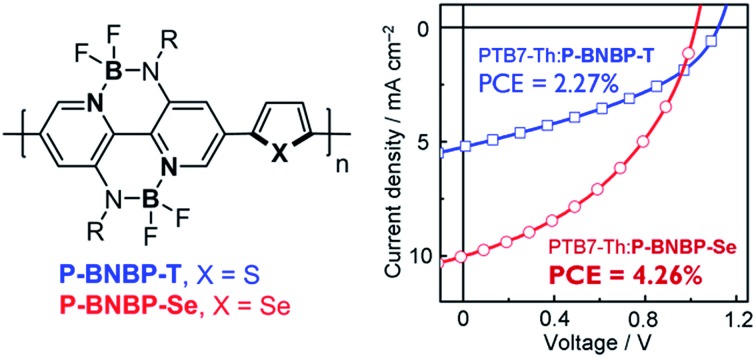
A new polymer acceptor based on the BNBP unit with an optimal LUMO energy level has been developed. The resulting all-polymer solar cells show high PCEs, remarkably high *V*_oc_ values and small photon energy losses.

## Introduction

All-polymer solar cells (all-PSCs), which utilize polymers as both the electron donor and electron acceptor, have attracted much attention recently because of their great advantages over conventional polymer/fullerene PSCs.^1^ These advantages include enhanced light absorption of polymer acceptors, low cost, and improved mechanical/thermal stability. Great progress in all-PSCs has been made by using absorption-complementary polymer donor/acceptors, optimizing the blend morphologies, or developing new polymer acceptors.^2^ However, the further development of all-PSCs is severely limited by the lack of excellent polymer acceptors.^3^ To date, only several specific polymer acceptors based on the naphthalene diimide (NDI) unit, perylenediimide (PDI) unit and B←N bridged thienylthiazole (BNTT) unit can work as polymer acceptors for efficient all-PSCs with power conversion efficiencies (PCEs) exceeding 4%.^4^,^5^

A key parameter for polymer acceptors is the lowest unoccupied molecular orbital (LUMO) energy level (*E*_LUMO_). In all-PSCs, the *E*_LUMO_ difference between the acceptor and donor (Δ*E*_LUMO_) is regarded as the driving force for the charge separation.^6^ The difference between the *E*_LUMO_ of the acceptor and the highest occupied molecular orbital (HOMO) energy level (*E*_HOMO_) of the donor is related to the open-circuit voltage (*V*_oc_) of all-PSCs.^6^ Therefore, to get a large Δ*E*_LUMO_ for effective charge separation and to maximize *V*_oc_, the *E*_LUMO_ of the polymer acceptor must be carefully optimized. The state-of-the-art polymer acceptors are the NDI-based conjugated polymers.^4^ Unfortunately, the *E*_LUMO_ of these polymers are fixed at *ca.* –3.85 eV and cannot be effectively tuned, leading to a low *V*_oc_ of the resulting all-PSCs. According to a study by Takimiya *et al.*,^7^ the fixed *E*_LUMO_ of NDI-based polymers are due to the localized LUMOs on the NDI units. The *E*_LUMO_ of the NDI-based conjugated polymers are determined by the NDI unit and are not affected by the copolymerization units. Thus, it is important but challenging to develop polymer acceptors with tunable *E*_LUMO_.

**Fig. 1:**
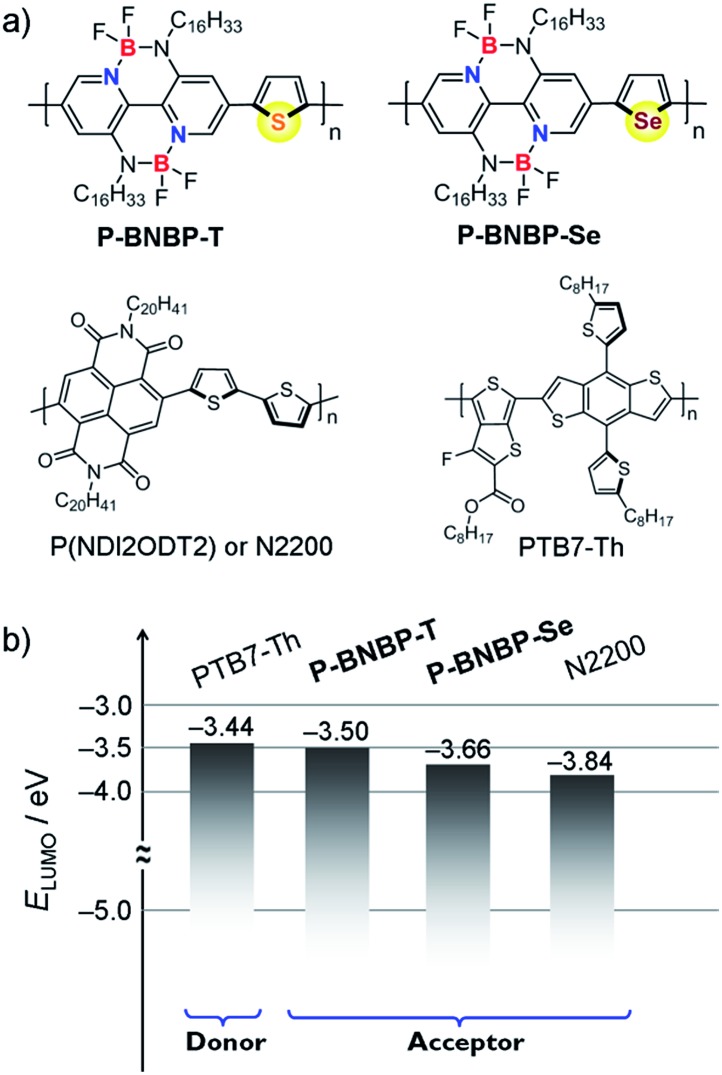
(a) Chemical structures of **P-BNBP-Se**, **P-BNBP-T**, **N2200** and **PTB7-Th** and (b) their LUMO energy level alignments.

Following our strategy to develop polymer acceptors using the B←N unit,^5^ we have reported a new electron-deficient building block based on the B←N unit, double B←N bridged bipyridine (BNBP), to develop a polymer acceptor.^8^ In this manuscript, we report that BNBP-based polymer acceptors show tunable *E*_LUMO_ because of their delocalized LUMOs over the polymer backbones. The *E*_LUMO_ of the copolymer of the BNBP unit and selenophene unit (**P-BNBP-Se**) is lower by 0.16 eV than that of the copolymer of the BNBP unit and thiophene unit (**P-BNBP-T**) (Fig. 1). As a result, the energy levels of **P-BNBP-Se** match well with the widely-used polymer donor, poly[(ethylhexyl-thiophenyl)-benzodithiophene-(ethylhexyl)-thienothiophene] (**PTB7-Th**).^9^ While the all-PSC device based on the **PTB7-Th**:**P-BNBP-T** blend shows a moderate PCE of 2.27%, the corresponding device with **P-BNBP-Se** as the acceptor exhibits a PCE as high as 4.26% with a remarkably high *V*_oc_ of 1.03 V. These results indicate that BNBP-based polymer acceptors have different electronic structures from those of the classical NDI-based polymer acceptors and that they can give all-PSCs with remarkably high *V*_oc_ values and high PCEs.

## Results and discussion

**Scheme 1:**
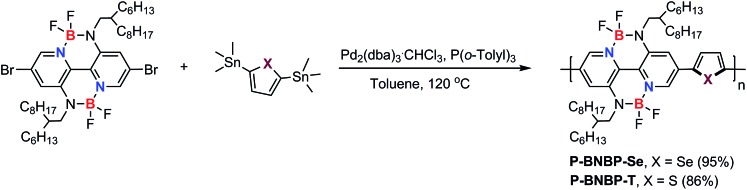
Synthetic route of **P-BNBP-Se** and **P-BNBP-T**.

Scheme 1 shows the synthetic route of **P-BNBP-Se** and **P-BNBP-T**. The three monomers were prepared following literature methods and the two polymers were synthesized in Stille-polymerization conditions.^8^ Their chemical structures are confirmed by ^1^H NMR and elemental analysis. According to gel permeation chromatography (GPC), with 1,2,4-trichlorobenzene as the eluent at 150 °C, the number-average molecular weight (*M*_n_) and polydispersity (PDI) are 26.3 kDa and 1.93 for **P-BNBP-Se** and 46.2 kDa and 1.81 for **P-BNBP-T**, respectively. According to the thermogravimetric analysis (TGA), **P-BNBP-T** and **P-BNBP-Se** show a good thermal stability with thermal decomposition temperatures (*T*_d_) of over 350 °C (ESI†). In addition, the two polymers show a good solubility in common organic solvents, including chlorobenzene (CB), chloroform (CHCl_3_) and *o*-dichlorobenzene (*o*-DCB).

**Fig. 2:**
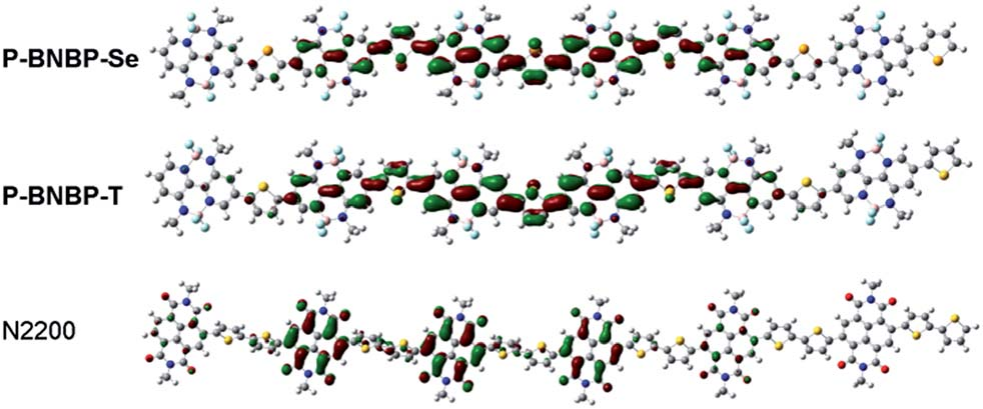
Kohn–Sham LUMOs of model compounds of **P-BNBP-Se**, **P-BNBP-T** and **N2200**, based on calculations at the B3LYP/6-31G* level.

To elucidate the molecular orbitals of the two polymers, density functional theory (DFT) calculations at the B3LYP/6-31G* level of theory were performed with the model compounds containing six repeating units with the long alkyl chains replaced by methyl groups.^10^ For comparison, we also show the DFT calculation result of the state-of-the-art polymer acceptor, (poly((*N*,*N*′-bis(2-octyldodecyl)-1,4,5,8-naphthalenedicarboximide-2,6-diyl)-*alt*-5,5′-(2,2-bithiophene))) (**N2200** or P(NDI2ODT2)) (Fig. 1a).^11^ As shown in Fig. 2, the calculated LUMO of the model compound of **N2200** is localized on the NDI units, indicating that its *E*_LUMO_ is determined by the NDI unit and cannot be effectively tuned by changing the co-monomer units. This is consistent with the DFT calculation and experimental results of NDI-based conjugated polymers in the literature.^4^,^11^ In contrast, the calculated LUMOs of the model compounds of **P-BNBP-Se**/**P-BNBP-T** are delocalized over the BNBP units and the selenophene/thiophene units. Therefore, the LUMO levels of BNBP-based polymers are determined by both the BNBP unit and the co-monomer unit. The LUMO levels of BNBP-based polymers should be effectively tuned by changing the co-monomer units.

**Fig. 3:**
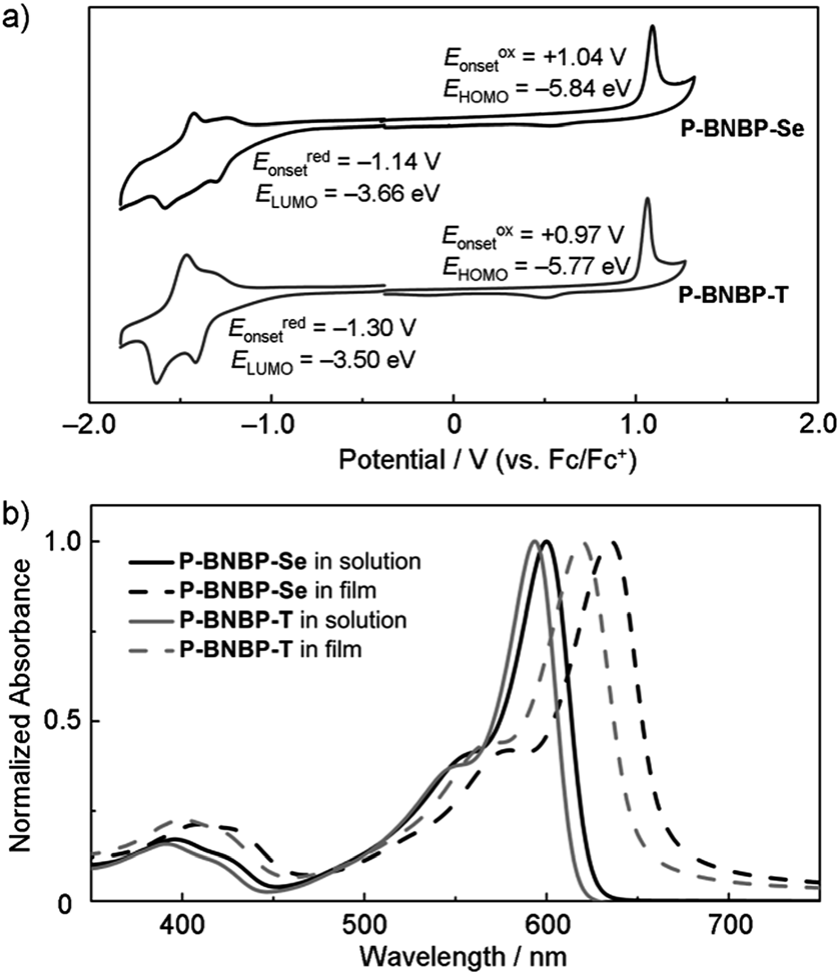
(a) Cyclic voltammogram of **P-BNBP-Se** and **P-BNBP-T** in thin films using a Ag/AgCl reference electrode, Fc = ferrocene; (b) UV/Vis absorption spectra of **P-BNBP-Se** and **P-BNBP-T** in *o*-DCB solutions and in thin films.

Cyclic voltammetry was employed to estimate the LUMO/HOMO energy levels of the two polymers (ESI†).^12^ As shown in Fig. 3a, **P-BNBP-Se** exhibits irreversible reduction and oxidation waves with onset potentials of *E*redonset = –1.14 V and *E*oxonset = +1.04 V, respectively. Accordingly, the *E*_LUMO/HOMO_ of **P-BNBP-Se** are estimated to be –3.66 eV/–5.84 eV (Table 1). Similarly, the *E*_LUMO/HOMO_ of **P-BNBP-T** are estimated to be –3.50 eV/–5.77 eV (Table 1). As reported previously, the model compound of the BNBP unit itself has an *E*_LUMO_ of –3.19 eV. The *E*_LUMO_ of the two BNBP-based polymers are much lower than that of the BNBP unit. Moreover, the *E*_LUMO_ of **P-BNBP-Se** is lower than that of **P-BNBP-T** by 0.16 eV. These results confirm that the LUMO levels of BNBP-based polymers can be effectively tuned by changing the co-monomer units. This is consistent with the delocalized LUMOs in the DFT calculation results. The lower-lying *E*_LUMO_ of **P-BNBP-Se** is attributed to the lower electronegativity of the Se atom (2.4) than the S atom (2.5) and the empty orbital of the Se atom.^13^

**Table 1:** Molecular weights, photophysical and electronic properties, and electron mobilities of **P-BNBP-Se** and **P-BNBP-T**

Polymer	*M*_n_ (kDa)	PDI	*λ*_abs_ ^*a*^ (nm)	*λ*_abs_ ^*b*^ (nm)	*ε* ^*b*^ (cm^–1^)	*E* opt g ^*b*^ (eV)	*E* ox onset ^*c*^ (V)	*E* red onset ^*c*^ (V)	*E*_HOMO_ ^*d*^ (eV)	*E*_LUMO_ ^*d*^ (eV)	*μ*_e_ (cm^2^ V^–1^ s^–1^)
**P-BNBP-Se**	26.3	1.93	600	635	1.49 × 10^5^	1.87	+1.04	–1.14	–5.84	–3.66	2.07 × 10^–4^
**P-BNBP-T**	46.0	2.01	593	622	1.45 × 10^5^	1.92	+0.97	–1.30	–5.77	–3.50	7.16 × 10^–5^

^*a*^Measured in *o*-DCB solution.

^*b*^Measured in thin film.

^*c*^Onset potential *vs.* Fc/Fc^+^.

^*d*^ *E*_HOMO/LUMO_ = –(4.80 + *E*oxonset/*E*redonset) eV.

Fig. 3b shows the absorption spectra of **P-BNBP-Se** and **P-BNBP-T** in dilute *o*-DCB solutions and in thin films. Both of the two polymers in solutions show broad absorption bands around *λ* = 580 nm. The absorption spectrum is slightly redshifted for **P-BNBP-Se** compared to **P-BNBP-T**. In thin film, **P-BNBP-Se** exhibits a maximum absorption at 635 nm, while **P-BNBP-T** shows the absorption peak at 622 nm. Both of the two films show high absorption coefficients (*ε*), suggesting their intense light absorption. According to the onset absorption wavelength in thin films, the optical band gaps (*E*_g_) of **P-BNBP-Se** and **P-BNBP-T** are estimated to be 1.87 eV and 1.92 eV, respectively. The electron mobilities (*μ*_e_) of **P-BNBP-Se** and **P-BNBP-T** were estimated using the space-charge-limited current (SCLC) method with the current density-voltage curves of the electron-only devices (device structure: ITO/PEIE/polymer/Ca/Al).^14^ The electron mobility of **P-BNBP-Se** (*μ*_e_ = 2.07 × 10^–4^ cm^2^ V^–1^ s^–1^) is higher than that of **P-BNBP-T** (*μ*_e_ = 7.16 × 10^–5^ cm^2^ V^–1^ s^–1^) (ESI†). The higher electron mobility of **P-BNBP-Se** is due to the stronger intermolecular interactions in Se-containing polymers because of the larger and more polarizable radii of the selenium atom than the sulfur atom. This is confirmed by the smaller π–π stacking distance of **P-BNBP-Se** (*d*_π–π_ = 3.77 Å) than that of **P-BNBP-T** (*d*_π–π_ = 3.81 Å) (ESI†). The electron mobility of **P-BNBP-Se** is comparable to the hole mobilities of typical polymer electron donors, which is very favourable for its application as a polymer electron acceptor in all-PSCs.

**Fig. 4:**
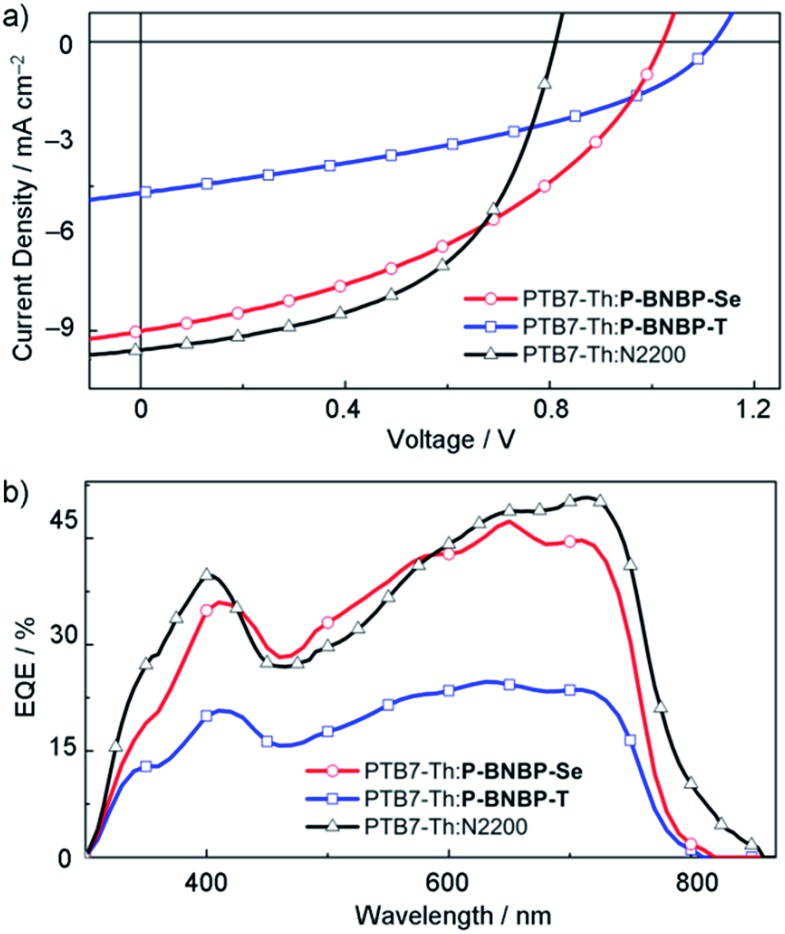
(a) *J*–*V* curves and (b) EQE spectra of the all-PSC devices based on the **PTB7-Th**:**P-BNBP-Se**, **PTB7-Th**:**P-BNBP-T** and **PTB7-Th**:**N2200** blends, respectively.

To investigate the application of **P-BNBP-Se** and **P-BNBP-T** as electron acceptors in all-PSCs, we select a widely-used polymer donor, **PTB7-Th**. All-PSC devices were fabricated with a configuration of ITO/PEDOT:PSS/**PTB7-Th**:**P-BNBP-Se** or **P-BNBP-T**/Ca/Al (ESI†). The active layer was spin-coated from the blend in *o*-DCB solution without any additives. Fig. 4 shows the current density–voltage (*J*–*V*) curves under AM 1.5G illumination (100 mW cm^–2^) and the external quantum efficiency (EQE) spectra of the optimal devices. The photovoltaic parameters are summarized in Table 2. The **PTB7-Th** : **P-BNBP-T** (3 : 1, w:w) device shows a PCE of 2.27% with a *V*_oc_ of 1.12 V, a short-circuit current density (*J*_sc_) of 5.24 mA cm^–2^ and a fill factor (FF) of 0.39. The device based on the **PTB7-Th** : **P-BNBP-Se** (2 : 1, w:w) blend exhibits a PCE of 4.26% with a *V*_oc_ of 1.03 V, a *J*_sc_ of 10.02 mA cm^–2^ and a FF of 0.42. This PCE value is comparable to that of the reference all-PSC device based on the **PTB7-Th** : **N2200** (1 : 1, w:w) blend from the chloroform solution (PCE = 4.57%), indicating that **P-BNBP-Se** is an excellent polymer acceptor. Compared with the device of **P-BNBP-T**, the device of **P-BNBP-Se** shows a slightly decreased *V*_oc_ and much increased *J*_sc_. The slightly decreased *V*_oc_ is attributed to the lower *E*_LUMO_ of **P-BNBP-Se** than that of **P-BNBP-T**. On the other hand, the *V*_oc_ of the **P-BNBP-Se** device is higher than that of the **N2200** device by 0.22 V (Table 2) because the *E*_LUMO_ of **P-BNBP-Se** is higher than that of **N2200**. The much increased *J*_sc_ of the **P-BNBP-Se** device than that of the **P-BNBP-T** device is in accordance with their EQE values (EQE_max_ = 0.47 for **P-BNBP-Se** and EQE_max_ = 0.25 for **P-BNBP-T**) (Fig. 4b). The *J*_sc_ calculated from the integration of the EQE spectra agrees well with the *J*_sc_ values obtained from the *J*–*V* scans within an error of 5%.

**Table 2:** Summary of the all-PSC device performance

Acceptor	*V*_oc_ (V)	*J*_sc_ (mA cm^–2^)	FF	PCE_max/ave_^*a*^ (%)	EQE	*E*_loss_ (eV)
**P-BNBP-Se**	1.03	10.02	0.42	4.26/4.11	0.47	0.56
**P-BNBP-T**	1.12	5.24	0.39	2.27/2.08	0.25	0.47
**N2200**	0.81	10.55	0.53	4.57/4.30	0.50	0.67

^*a*^The average PCE value is calculated from eight devices.

**Fig. 5:**
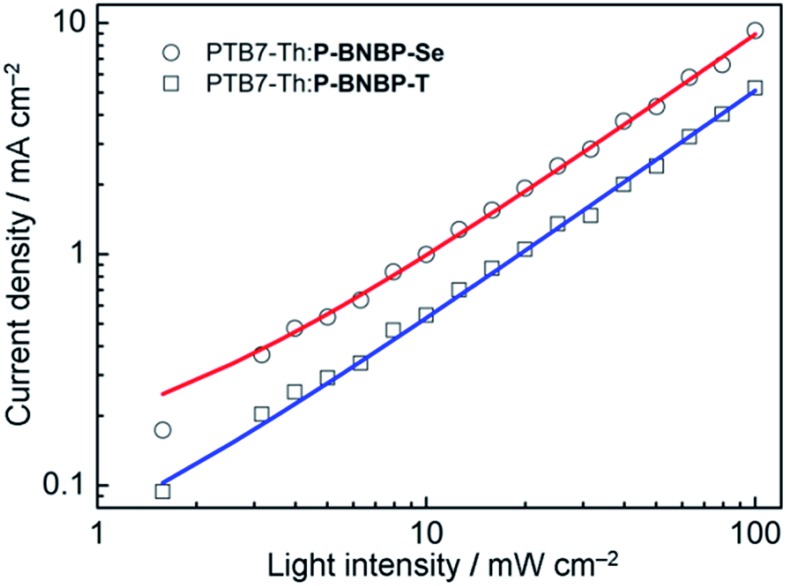
Short-circuit current density (*J*_sc_) *versus* light intensity (*P*_light_) data and power-law (*J*_sc_ ∝ *P*_light_^*α*^) fittings for the all-PSC devices.

The charge carrier mobilities of the two blends were investigated using the SCLC method with the electron-only and hole-only devices (ESI†).^14^ The electron mobility and hole mobility (*μ*_h_) of the **PTB7-Th**:**P-BNBP-Se** blend are estimated to be 3.34 × 10^–5^ cm^2^ V^–1^ s^–1^ and 2.38 × 10^–4^ cm^2^ V^–1^ s^–1^, respectively. In comparison, the **PTB7-Th**:**P-BNBP-T** blend exhibits a *μ*_e_ = 5.96 × 10^–6^ cm^2^ V^–1^ s^–1^ and *μ*_h_ = 7.28 × 10^–4^ cm^2^ V^–1^ s^–1^, respectively. The higher electron mobility and the balanced electron/hole mobilities of the **PTB7-Th**:**P-BNBP-Se** blend are due to the enhanced electron mobility of **P-BNBP-Se**. We also investigated the bimolecular charge recombination in the all-PSC devices using the light-intensity dependence of the *J*–*V* curves (Fig. 5). The *J*_sc_ follows a power-law dependence on the illumination intensity (*J*_sc_ ∝ *P*_light_^*α*^), where *P*_light_ is light intensity and *α* is the calculated power-law exponent. The *α* values are 0.93 for the **PTB7-Th**:**P-BNBP-Se** device and 0.94 for the **PTB7-Th**:**P-BNBP-T** device, which are close to unity, suggesting that the bimolecular charge recombination is weak in the two devices at a short circuit condition.^15^ Both the weak bimolecular recombination and the high and balanced electron/hole mobilities of **PTB7-Th**:**P-BNBP-Se** can explain its excellent device performance.

**Fig. 6:**
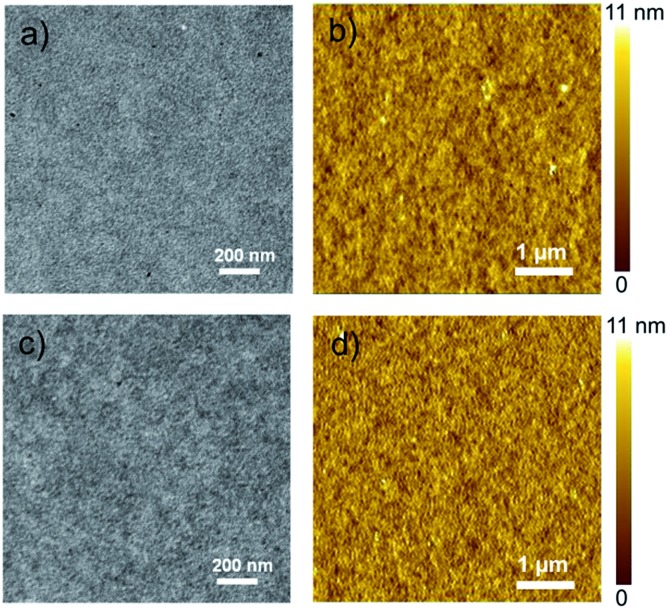
The TEM images and the AFM height images of ((a) and (b)) the **PTB7-Th**:**P-BNBP-Se** blend and ((c) and (d)) the **PTB7-Th**:**P-BNBP-T** blend, respectively.

The morphologies of the **PTB7-Th**:**P-BNBP-Se** and **PTB7-Th**:**P-BNBP-T** blends were characterized by transmission electron microscopy (TEM) and atomic force microscopy (AFM). As shown in Fig. 6, the TEM images exhibit similar nano/microstructures without large-size aggregation. The AFM images of the two blends similarly reveal smooth surface morphologies with the same root-mean-square (RMS) roughness of 1.47 nm and domain sizes of around 20–40 nm. The phase separation morphologies of the two blends are beneficial for good all-PSC devices.

In organic photovoltaics (OPVs), the Δ*E*_LUMO_ of the donor and acceptor is regarded as the driving force for charge separation. The Δ*E*_LUMO_ should be larger than a specific value for efficient charge separation. If Δ*E*_LUMO_ is too large, there is a lot of energy loss in the charge separation process, leading to a low *V*_oc_ because the *V*_oc_ of OPVs is related to the difference between the *E*_HOMO_ of the donor and *E*_LUMO_ of the acceptor.^6^ In our previous report, an all-PSC device based on the PTB7:**P-BNBP-T** blend (Δ*E*_LUMO_ = 0.19 eV) showed a good PCE of 3.38%.^8^ As shown in Fig. 1b, the Δ*E*_LUMO_ is only 0.06 eV for **PTB7-Th**:**P-BNBP-T**, and thus the all-PSC device shows a high *V*_oc_ but produces a low PCE due to the insufficient charge separation.4*g* As the *E*_LUMO_ of **P-BNBP-Se** is lower than that of **P-BNBP-T**, the Δ*E*_LUMO_ for **PTB7-Th**:**P-BNBP-Se** is increased to 0.22 eV and ensures an efficient charge separation, resulting in higher *J*_sc_ and PCE values. Moreover, due to the suitable *E*_LUMO_ of **P-BNBP-Se**, the **PTB7-Th**:**P-BNBP-Se** device produces a high *V*_oc_ of 1.03 V, which is higher than that of the **PTB7-Th**:**N2200** device by 0.22 V. These results indicate that the suitable *E*_LUMO_ of **P-BNBP-Se** plays an important role in enhancing the all-PSCs device performance.

It is worthy to note the remarkably low photon energy losses (*E*_loss_) of the all-PSCs based on **P-BNBP-Se** and **P-BNBP-T**. *E*_loss_ is defined as the difference between the lowest optical bandgap of the donor/acceptor and the *eV*_oc_ of the organic photovoltaic (OPV) device (*E*_loss_ = *E*_g_ – *eV*_oc_).^16^ Typically, OPVs have large *E*_loss_ values of 0.7–1.0 eV. It has been proposed that the lowest attainable *E*_loss_ of OPVs is 0.6 eV, despite several exceptional examples.^17^ As listed in Table 2, the *E*_loss_ for the device of **PTB7-Th**:**P-BNBP-Se** and **PTB7-Th**:**P-BNBP-T** is 0.56 eV and 0.47 eV, respectively. To our best knowledge, the *E*_loss_ of 0.47 eV is the lowest one for OPVs reported so far. A small *E*_loss_ is always observed for all-PSCs with BNBP-based polymers as electron acceptors and the exact reason is as yet unknown. We speculate that the small *E*_loss_ is related to the high-lying LUMO levels of the BNBP-based polymers.

## Conclusions

In summary, we have developed a polymer acceptor based on the BNBP unit and selenophene unit with an optimal *E*_LUMO_ to simultaneously enable charge separation and maximize *V*_oc_. BNBP-based polymers have delocalized LUMOs over the polymer backbones, so their *E*_LUMO_ can be tuned by changing the comonomer unit. The *E*_LUMO_ of **P-BNBP-Se** is lower by 0.16 eV than that of **P-BNBP-T** and consequently matches well with that of the polymer donor, **PTB7-Th**. While the all-PSC device based on **PTB7-Th**:**P-BNBP-T** shows a moderate PCE of 2.27%, the corresponding device with **P-BNBP-Se** as the acceptor exhibits a PCE as high as 4.26%. Moreover, the device of **P-BNBP-Se** shows a *V*_oc_ of up to 1.03 V and *E*_loss_ as small as 0.56 eV. These results indicate that BNBP-based polymer acceptors have different electronic structures from those of classical NDI-based polymer acceptors and that they can give all-PSCs with remarkably high *V*_oc_ values and high PCEs.

## Experimental section

### Synthesis of **P-BNBP-Se**

The dibromo-substituted BNBP monomer was synthesized according to the previous report.^8^ The starting materials of the dibromo-substituted BNBP monomer (220 mg, 0.248 mmol), 2,5-bis(trimethylstannyl)selenophene (114.1 mg, 0.248 mmol), Pd_2_(dba)_3_·CHCl_3_ (4.5 mg, 0.0050 mmol) and P(*o*-tolyl) (12.1 mg, 0.04 mmol) were placed in a two-necked flask under argon, and then dried toluene (11 mL) was added. After the mixture was stirred at 115 °C for 48 h, an end-capping reaction was carried out by adding 2,5-bis(trimethylstannyl)selenophene (3 mg) and then bromobenzene (200 mg). After cooling, the resulting organic phase was extracted with CHCl_3_ (150 mL) and washed with water. After the solvents were removed, the residue was dispersed in methanol and the precipitate was collected. The obtained dark solid was dispersed in acetonitrile, and was collected and dried in a vacuum overnight. Yield: 213 mg (95%). ^1^H NMR (400 MHz, CDCl_3_, 25 °C): *δ* 8.41 (s, 1H), 7.76 (s, 1H), 7.66 (s, 1H), 3.63 (br, 2H), 1.85 (br, 1H), 1.42–1.27 (br, 24H), 0.85 (br, 6H). GPC (TCB, polystyrene standard, 150 °C): *M*_n_ = 26 300, PDI = 1.93. Anal. calcd for C_46_H_72_B_2_F_4_N_4_Se: C, 64.42; H, 8.46; B, 2.52; F, 8.86; N, 6.53; Se, 9.21. Found: C, 64.25; H, 8.58; N, 6.65; Se, 9.05.

### Synthesis of **P-BNBP-T**

The starting materials of the dibromo-substituted BNBP monomer (150 mg, 0.166 mmol), 2,5-bis(trimethylstannyl)thiophene (68.5 mg, 0.166 mmol), Pd_2_(dba)_3_·CHCl_3_ (3.5 mg, 0.0033 mmol) and P(*o*-tolyl) (8.1 mg, 0.027 mmol) were placed in a two-necked flask under argon, and then dried toluene (4 mL) was added. After the mixture was stirred at 115 °C for 50 h, an end-capping reaction was carried out by adding 2,5-bis(trimethylstannyl)thiophene (3 mg) and then bromobenzene (200 mg). After cooling, the resulting organic phase was extracted with CHCl_3_ (150 mL) and washed with water. After the solvents were removed, the residue was dispersed in methanol and the precipitate was collected. The obtained dark solid was dispersed in acetonitrile, and was collected and dried in a vacuum overnight. Yield: 116.5 mg (86%). ^1^H NMR (400 MHz, CDCl_3_, 25 °C): *δ* 8.47 (s, 1H), 7.74 (s, 1H), 7.60 (s, 1H), 3.64 (br, 2H), 1.87 (br, 1H), 1.43–1.25 (br, 24H), 0.83 (br, 6H). GPC (TCB, polystyrene standard, 150 °C): *M*_n_ = 46 200, PDI = 1.81. Anal. calcd for C_46_H_72_B_2_F_4_N_4_S: C, 68.14; H, 8.95; B, 2.67; F, 7.37; N, 6.91; S, 3.95. Found: C, 67.83; H, 8.82; N, 6.75; S, 4.05.

## Supplementary Material

Supplementary information

## References

[cit1] Kim T., Kim J.-H., Kang T. E., Lee C., Kang H., Shin M., Wang C., Ma B., Jeong U., Kim T.-S., Kim B. J. (2015). Nat. Commun..

[cit2] Gao L., Zhang Z.-G., Xue L., Min J., Zhang J., Wei Z., Li Y. (2016). Adv. Mater..

[cit3] Benten H., Mori D., Ohkita H., Ito S. (2016). J. Mater. Chem. A.

[cit4] Zhou N., Dudnik A. S., Li T. I. N. G., Manley E. F., Aldrich T. J., Guo P., Liao H.-C., Chen Z., Chen L. X., Chang R. P. H., Facchetti A., de la Cruz M. O., Marks T. J. (2016). J. Am. Chem. Soc..

[cit5] Zhao R., Dou C., Xie Z., Liu J., Wang L. (2016). Angew. Chem., Int. Ed..

[cit6] Street R. A. (2016). Adv. Mater..

[cit7] Takimiya K., Osaka I., Nakano M. (2014). Chem. Mater..

[cit8] Dou C., Long X., Ding Z., Xie Z., Liu J., Wang L. (2016). Angew. Chem., Int. Ed..

[cit9] He Z., Zhong C., Su S., Xu M., Wu H., Cao Y. (2012). Nat. Photonics.

[cit10] DFT calculations were performed using Gaussian 09 program: M. J. Frisch, *et al.*, *Gaussian 09, revision A.02*, Gaussian, Inc., Wallingford, CT, 2009. For details, see ESI.

[cit11] Steyrleuthner R., Schubert M., Howard I., Klaumünzer B., Schilling K., Chen Z., Saalfrank P., Laquai F., Facchetti A., Neher D. (2012). J. Am. Chem. Soc..

[cit12] Ding Z., Miao Z., Xie Z., Liu J. (2016). J. Mater. Chem. A.

[cit13] Meng D., Sun D., Zhong C., Liu T., Fan B., Huo L., Li Y., Jiang W., Choi H., Kim T., Kim J. Y., Sun Y., Wang Z., Heeger A. J. (2016). J. Am. Chem. Soc..

[cit14] Blom P. W. M., de Jong M. J. M., van Munster M. G. (1997). Phys. Rev. B: Solid State.

[cit15] Kywa A. K. K., Wang D. H., Gupta V., Leong W. L., Ke L., Bazan G. C., Heeger A. J. (2013). ACS Nano.

[cit16] Janssen R. A. J., Nelson J. (2013). Adv. Mater..

[cit17] Kawashima K., Tamai Y., Ohkita H., Osaka I., Takimiya K. (2015). Nat. Commun..

