# Supplementary material for: A polymer acceptor with an optimal LUMO energy level for all-polymer solar cells
Source: Chem Sci. 2016 Jun 14;7(9):6197–202. doi: 10.1039/c6sc01756h (PMC6024205; doi:10.1039/c6sc01756h)
Supplement: Supplementary file 1 [file SC-007-C6SC01756H-s001.pdf]

Electronic Supplementary Material (ESI) for Chemical Science.
This journal is © The Royal Society of Chemistry 2016

Supporting Information

for

A Polymer Acceptor with An Optimal LUMO Energy Level for All-Polymer Solar Cells

Zicheng Ding,[†a] Xiaojing Long,[†ab] Chuandong Dou,[*a] Jun Liu[*a] and Lixiang Wang[a]

[a]*State Key Laboratory of Polymer Physics and Chemistry, Changchun Institute of Applied Chemistry, Chinese Academy of Sciences, Changchun 130022, People's Republic of China.*
[b]*University of Chinese Academy of Sciences, Beijing 100864, People's Republic of China.*
[†]*Dr. Z. Ding and X. Long contributed equally to this work.*
Email: chuandong.dou@ciac.ac.cn; liujun@ciac.ac.cn.

Contents

1. Experimental details

2. Thermal properties

3. Morphological properties

4. Theoretical calculations

5. Photophysical properties

6. Electrochemical properties

7. All-PSC device fabrications and measurements

8. References

9. ^{1}H NMR spectra

1. Experimental details

General. ^{1}H NMR spectra were measured with a Bruker AV-400 (400 MHz) spectrometer in $CDCl_3$ at 25 °C. Chemical shifts are reported in δ ppm using $CHCl_3$ (7.26 ppm) for ^{1}H NMR as an internal standard. Elemental analysis was performed on a VarioEL elemental analyzer. The molecular weight of the polymer was determined by gel permeation chromatography (GPC) on a PL-GPC 220-type at the temperature of 150 °C. 1,2,4-Trichlorobenzene (TCB) was used as the eluent and monodisperse polystyrene was used as the standard. UV/Vis absorption spectra were measured with a Shimadzu UV-3600 spectrometer in spectral grade solvents. Thermal analyses were performed on a Perkin-Elmer 7 instrument under nitrogen flow at a heating rate of 10 °C min^{-1}. The transmission electron microscope (TEM) image was obtained with a JEM-1011 (JEOL Co., Japan) operated at an accelerating voltage of 100 kV. Atomic force microscopy (AFM) was performed with a SPA300HV (Seiko Instruments, Inc., Japan) in tapping mode. GI-XRD data was obtained on a Bruker D8 Discover reflector (Cu Kα, λ = 1.54056 Å) under 40 kV and 40 mA tube current. Cyclic voltammetry (CV) was performed on an CHI660a electrochemical workstation using Bu_4NClO_4 (0.1 M) in acetonitrile as electrolyte solution and ferrocene as an internal reference at a scan rate of 100 mV s^{-1}. The CV cell consisted of a glassy carbon electrode, a Pt wire counter electrode, and a Ag/AgCl reference electrode. The polymer was casted on the working electrode for measurements. The redox potentials were calibrated with ferrocene as an internal standard. The highest occupied molecular orbital (HOMO) and the lowest unoccupied molecular orbital (LUMO) energy levels of the materials were estimated by the equations: $E_{HOMO/LUMO} = -(4.80 + E_{onset}^{ox}/E_{onset}^{red})$ eV. All reactions were performed under argon atmosphere. Commercially available solvents and reagents were used without further purification unless otherwise mentioned. Toluene was dried using sodium before use. BNBP monomer was prepared according to the literature method.[1] $Pd_2(dba)_3{\cdot}CHCl_3$ was fully purified according to the literature method.[2]

2. Thermal properties

The thermal property of **P-BNBP-Se** was determined by thermogravimetric analysis (TGA). TGA analysis showed that it had good thermal stability with decomposition temperature at 5% weight loss of 400 °C under N_2.

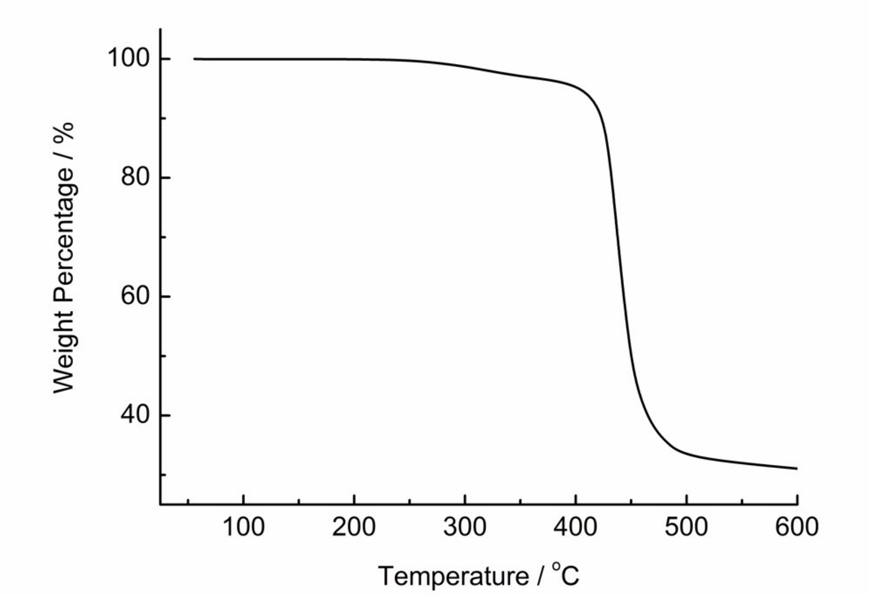

Figure S1. TGA curve of **P-BNBP-Se**.

3. Morphological properties

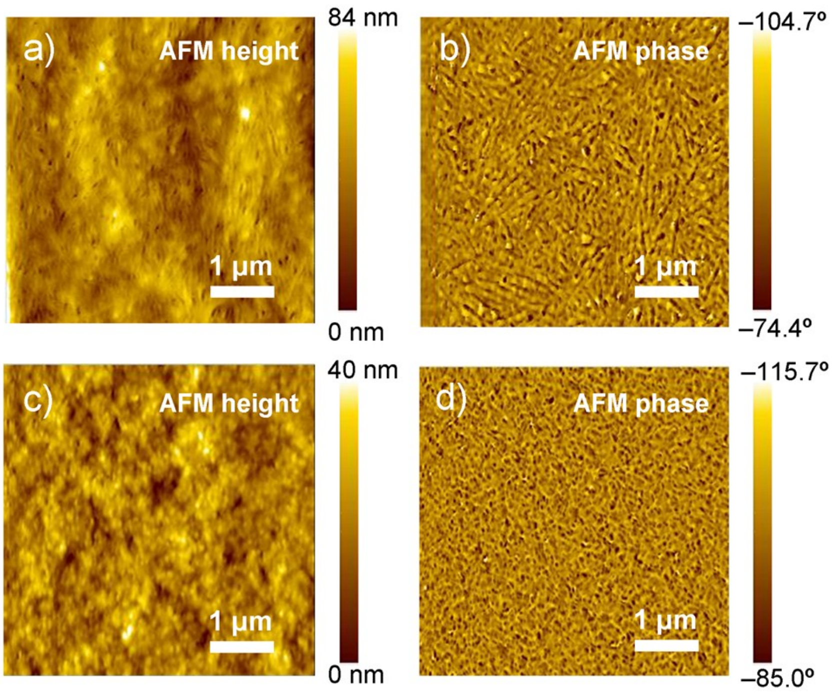

Figure S2. The AFM height and phase images of the drop-cast films: a) and b) **P-BNBP-Se** and c) and d) **P-BNBP-T** from their *o*-DCB solutions, respectively.

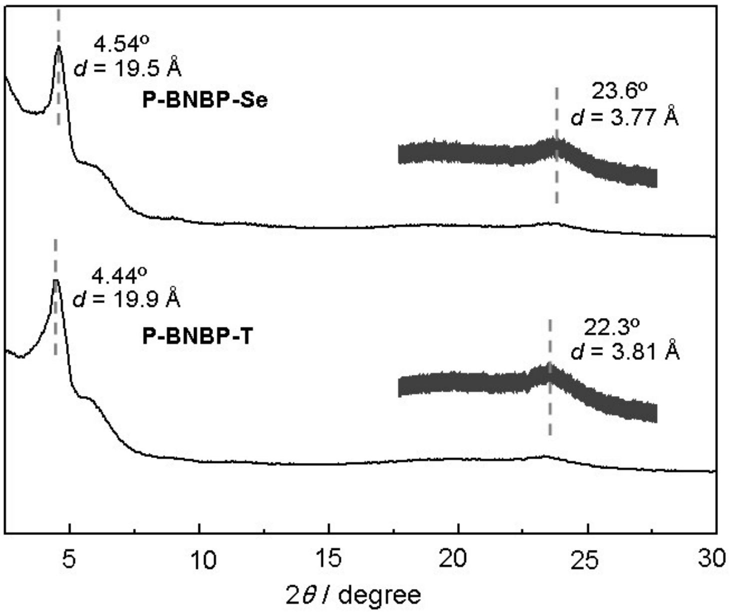

Figure S3. Grazing incidence X-ray diffraction (GI-XRD) curves of the drop-cast films of **P-BNBP-Se** and **P-BNBP-T**. The ordered lamellar structures are observed with the layer spacing and π–π stacking distances of 19.5 Å and 3.77 Å for **P-BNBP-Se** and 19.9 Å and 3.81 Å for **P-BNBP-T**, respectively.

4. Theoretical calculations

All calculations were performed with the Gaussian 09 program.[3] The structural optimizations and DFT calculations of the model compounds were performed using Gaussian 09 program at the B3LYP/6-31G* level of theory. The model compounds contain six repeating units with the long alkyl chains replaced by the methyl groups on nitrogen atoms.

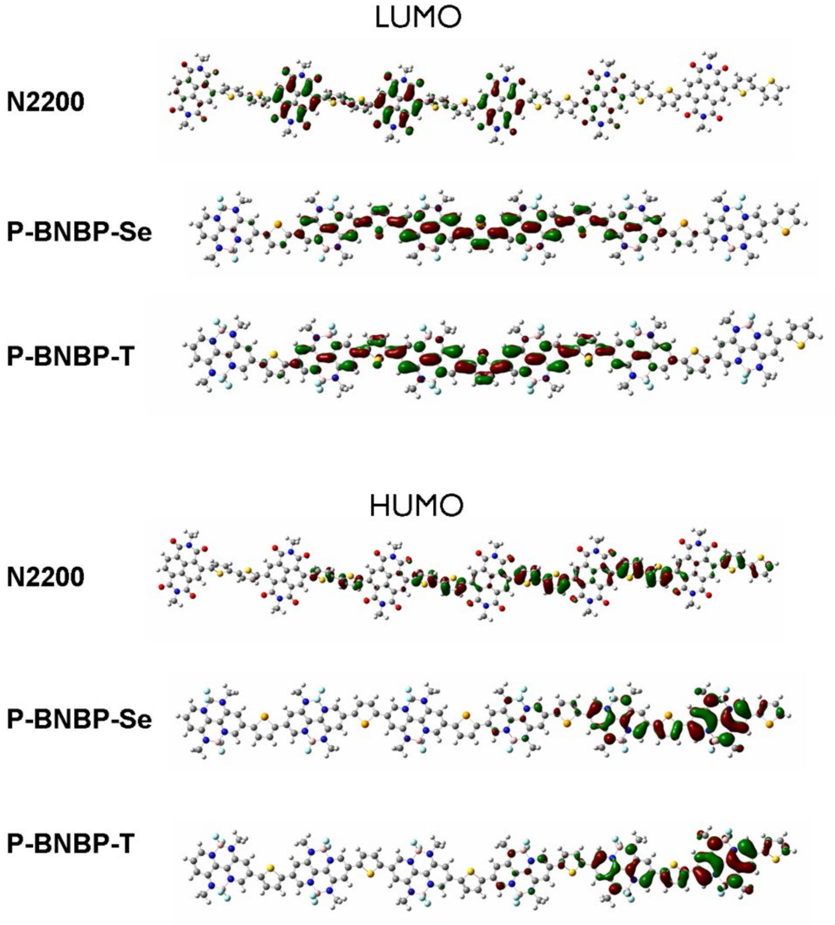

Figure S4. Comparison of the electronic structures of the model compounds of BNBP-based polymers and N2200.

5. Photophysical properties

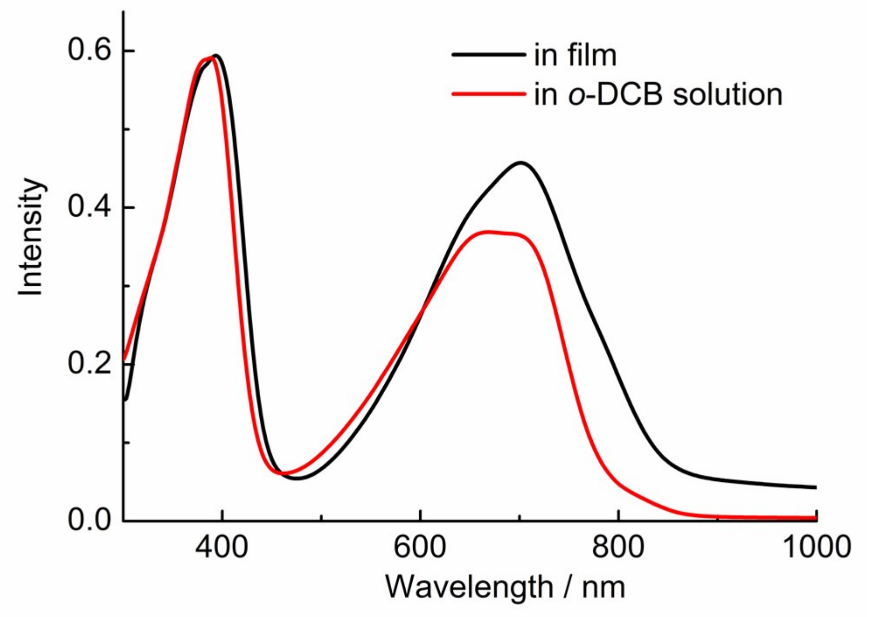

Figure S5. UV/Vis absorption spectra of N2200 in *o*-DCB solution and in thin film. The film absorption coefficient at 701 nm of the N2200 film is 3.78×10^4 cm^{-1}, which is smaller than those of **P-BNBP-Se** and **P-BNBP-T**.

6. Electrochemical properties

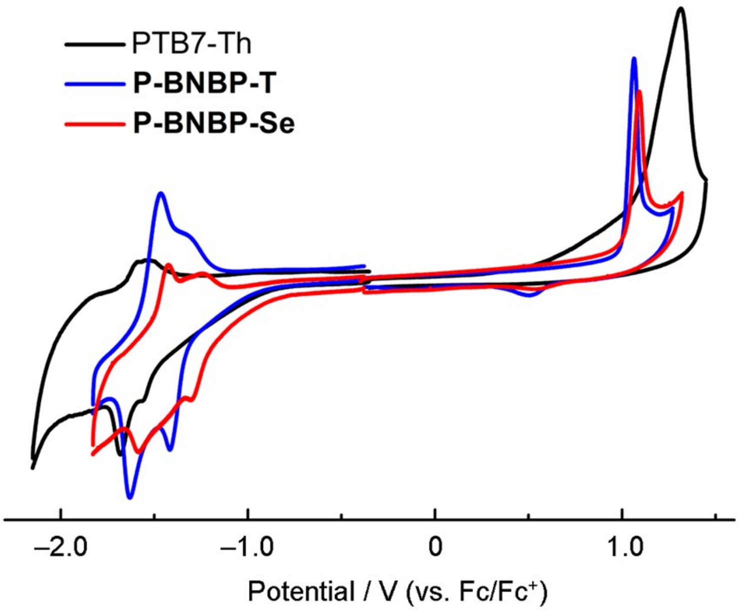

Figure S6. Cyclic voltammogram spectra of PTB7-Th, **P-BNBP-Se** and **P-BNBP-T** in films, using a Ag/AgCl reference electrode. Fc = ferrocene. According to the onset potential of reduction and oxidation waves in the cyclic voltammogram of PTB7-Th (E_{onset}^{red} = –1.36 V and E_{onset}^{ox} = +0.54 V), its LUMO/HOMO energy levels are estimated to be –3.44 eV/–5.34 eV.

7. All-PSC device fabrications and measurements

PTB7-Th and N2200 were purchased from 1-material Chemscitech Inc. and Polyera Inc., respectively. Indium tin oxide (ITO) glass substrates were cleaned by sequential ultrasonication in detergent, deionized water, acetone, and isopropyl alcohol, followed by dried at 120 °C for 30 min and treated with UV-ozone for 25 min. Then PEDOT:PSS (Clevios VP Al 4083 from H. C. Starck Inc.) was spin-coated on the ITO glass substrates at 5000 rpm for 40 s to give a thickness of 40 nm, followed by baking at 120 °C for 30 min. The substrates were transferred to a nitrogen-filled glove box. The blend was spin-coated onto the PEDOT:PSS layer to produce the active layer (90–110 nm). The blend ratios of the devices with the best performance are shown. The blend ratio is 2:1 (*w/w*) for PTB7-Th:**P-BNBP-Se** in *o*-DCB solution. For PTB7-Th:**P-BNBP-T** in *o*-DCB solution**,** the blend ratio is 3:1 (*w/w*). For PTB7-Th:N2200 in chloroform solution, the blend ratio is 1:1 (*w/w*). The active layer of PTB7-Th/**P-BNBP-Se** blend was annealed at 170 °C for 10 min, while the active layers of other two blends were annealed at 80 °C for 10 min. Finally, the active layer was transferred to a vacuum chamber, and Ca (20 nm) and Al (100 nm) were deposited by thermal evaporation at the pressure of about 2 × 10^{-4} Pa. The active area of each device was 8 mm^2. The current density (*J*−*V*) curves of the PSC devices were measured using a computer-controlled Keithley 2400 source meter under 100 mW cm^{-2} AM 1.5G simulated solar light illumination provided by a XES-40S2-CE Class Solar Simulator (SAN-EI Electric Co., Ltd., Japan). The EQE spectrum was measured using a Solar Cell Spectral Response Measurement System QE-R3011 (Enlitech Co., Ltd.). The light intensity at each wavelength was calibrated using a calibrated monosilicon diode.

The electron/hole mobilities of the films were measured using the space-charge-limited current (SCLC) method. The electron-only and hole-only device structures for devices are ITO/PEIE/Active layer/Ca/Al and ITO/PEDOT:PSS/Active layer/MoO_3/Ag, respectively. The current-voltage curves in the range of 0–10 V were recorded using a computer-controlled Keithley 2400 source meter, and the results were fitted to a space-charge limited function:

$$J = \frac{9}{8}\varepsilon_r\varepsilon_0\mu\frac{V^2}{L^3}\exp\left(0.89\beta\frac{\sqrt{V}}{\sqrt{L}}\right)$$

where J is the current density, ε_0 is the permittivity of free space, ε_r is the relative permittivity of 2.3 for **PBNBP-Se** and **P-BNBP-T**, μ is the zero-field mobility, V is the potential across the device (V = $V_{applied}$–V_{bias}–V_{series}), L is the thickness of

active layer, and β is the field-activation factor. The series and contact resistance (V_{series}) of the device (10–15 Ω) were measured using blank devices of ITO/PEIE/Ca/Al or ITO/PEDOT:PSS/MoO_3/Ag.

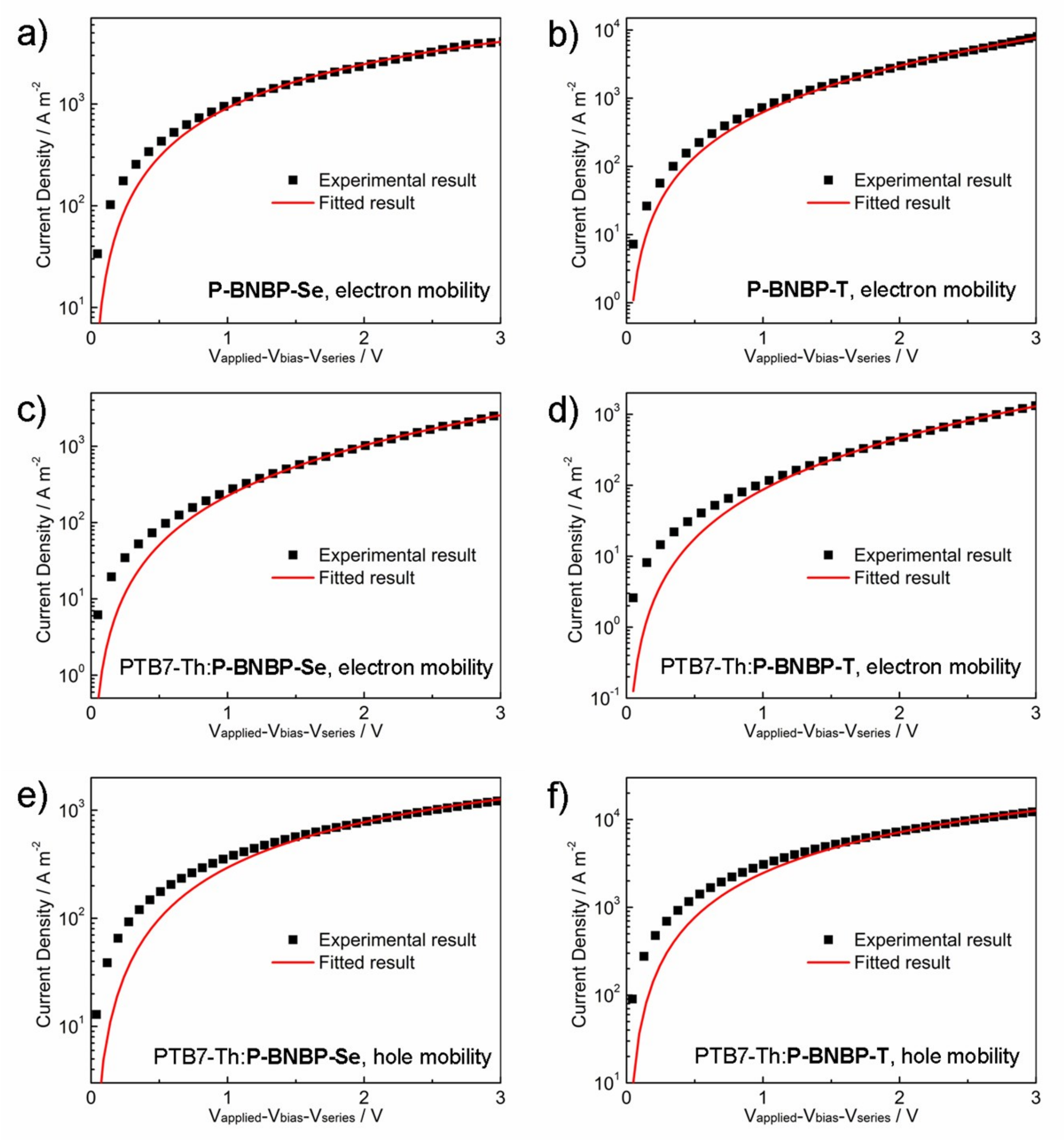

Figure S7. Space-charge-limited current (SCLC) fittings of the devices based on the prime films and the blend films. The field-activation factor β ($cm^{0.5}$ $V^{-0.5}$) and Adj. R-square are a) –2.60 × 10^{-4} and 0.99643, b) 1.32 × 10^{-4} and 0.99846, c) 1.02 × 10^{-4} and 0.99844, d) 2.07 × 10^{-4} and 0.99859, e) –3.41 × 10^{-4} and 0.99569, and f) –2.27 × 10^{-4} and 0.99768, respectively.

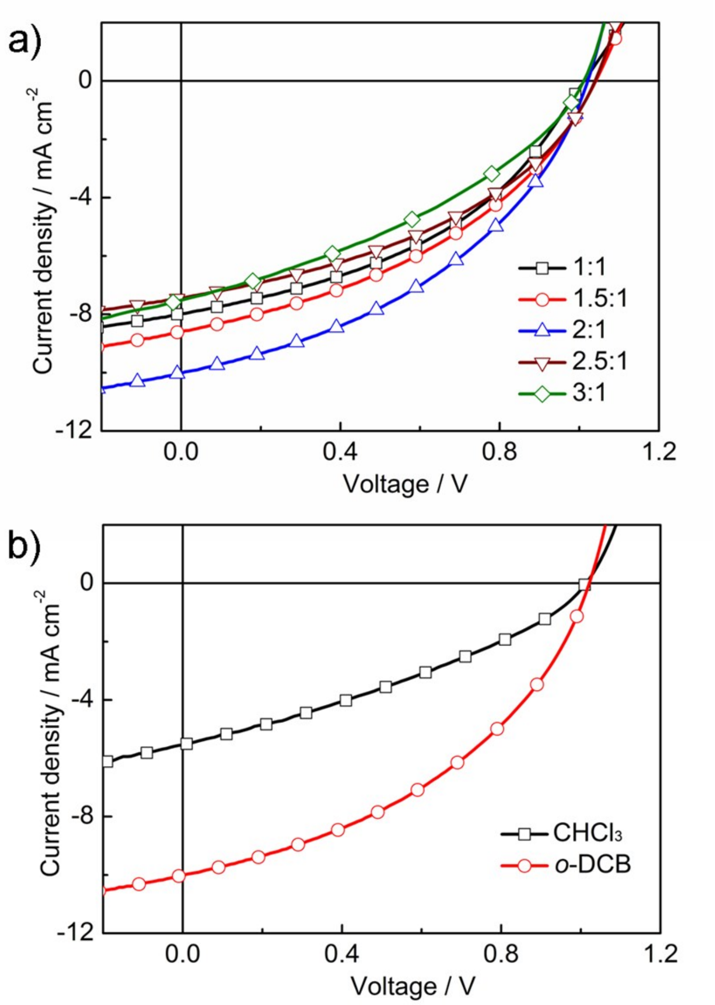

Figure S8. *J–V* curves of the all-PSC devices based on the PTB7-Th:**P-BNBP-Se** blends with a) different D:A ratios in *o*-DCB solutions and b) 2:1 D:A ratio in $CHCl_3$ and *o*-DCB solutions.

Table S1. Summary of the PTB7-Th:**P-BNBP-Se** all-PSC device performance.

D:A (*w*:*w*)	V_{oc} (V)	J_{sc} (mA cm^{-2})	FF	$PCE_{max/ave}$[c] (%)
1:1[a]	1.01	7.98	0.42	3.39 (3.18)
1.5:1[a]	1.04	8.60	0.40	3.61 (3.50)
2:1[a]	1.03	10.02	0.42	4.26 (4.11)
2.5:1[a]	1.04	7.43	0.41	3.21 (3.05)
3:1[a]	1.02	7.52	0.36	2.78 (2.65)
2:1[b]	1.01	5.55	0.33	1.85 (1.75)

[a]The blend from *o*-DCB solution. [b]The blend from $CHCl_3$ solution. [c]The average PCE value is calculated from eight devices.

8. References

[1] Dou, C.; Long, X.; Ding, Z.; Xie, Z.; Liu, J.; Wang, L. *Angew. Chem. Int. Ed.* **2016**, *55*, 1436.

[2] Zalesskiy, S. S.; Ananikov, V. P. *Organometallics* **2012**, *31*, 2302.

[3] Gaussian 09 (Revision A.02), Frisch, M. J.; Trucks, G. W.; Schlegel, H. B.; Scuseria, G.E.; Robb, M. A.; Cheeseman, J. R.; Scalmani, G.; Barone, V.; Mennucci, B.; Petersson, G. A.; Nakatsuji, H.; Caricato, M.; Li, X.; Hratchian, H. P.; Izmaylov, A. F.; Bloino, J.; Zheng, G.; Sonnenberg, J. L.; Hada, M.; Ehara, M.; Toyota, K.; Fukuda, R.; Hasegawa, J.; Ishida, M.; Nakajima, T.; Honda, Y.; Kitao, O.; Nakai, H.; Vreven, T.; Montgomery, Jr., J. A.; Peralta, J. E.; Ogliaro, F.; Bearpark, M.; Heyd, J. J.; Brothers, E.; Kudin, K. N.;Staroverov, V. N.; Kobayashi, R.; Normand, J.; Raghavachari, K.; Rendell, A.; Burant, J. C.; Iyengar, S. S.; Tomasi, J.; Cossi, M.; Rega, N.; Millam, J. M.; Klene, M.; Knox, J. E.; Cross, J. B.; Bakken, V.; Adamo, C.; Jaramillo, J.; Gomperts, R.; Stratmann, R. E.; Yazyev, O.; Austin, A. J.; Cammi, R.; Pomelli, C.; Ochterski, J. W.; Martin, R. L.; Morokuma, K.; Zakrzewski, V. G.; Voth, G. A.; Salvador, P.; Dannenberg, J. J.; Dapprich,S.; Daniels, A. D.; Farkas, O.; Foresman, J. B.; Ortiz, J. V.; Cioslowski, J.; Fox, D. J. Gaussian, Inc., Wallingford CT, **2009**.

9. ^{1}H NMR spectra

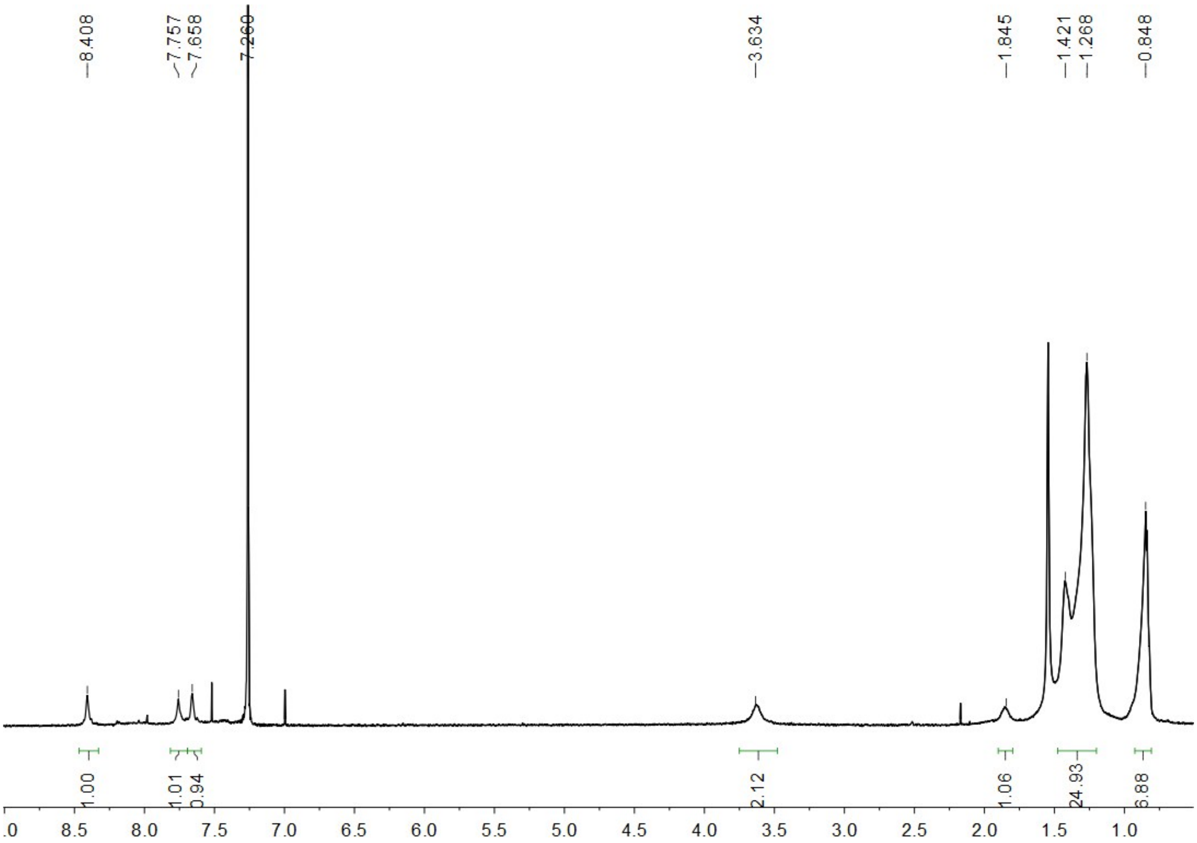

Figure S9. ^{1}H NMR spectrum of **P-BNBP-Se**.

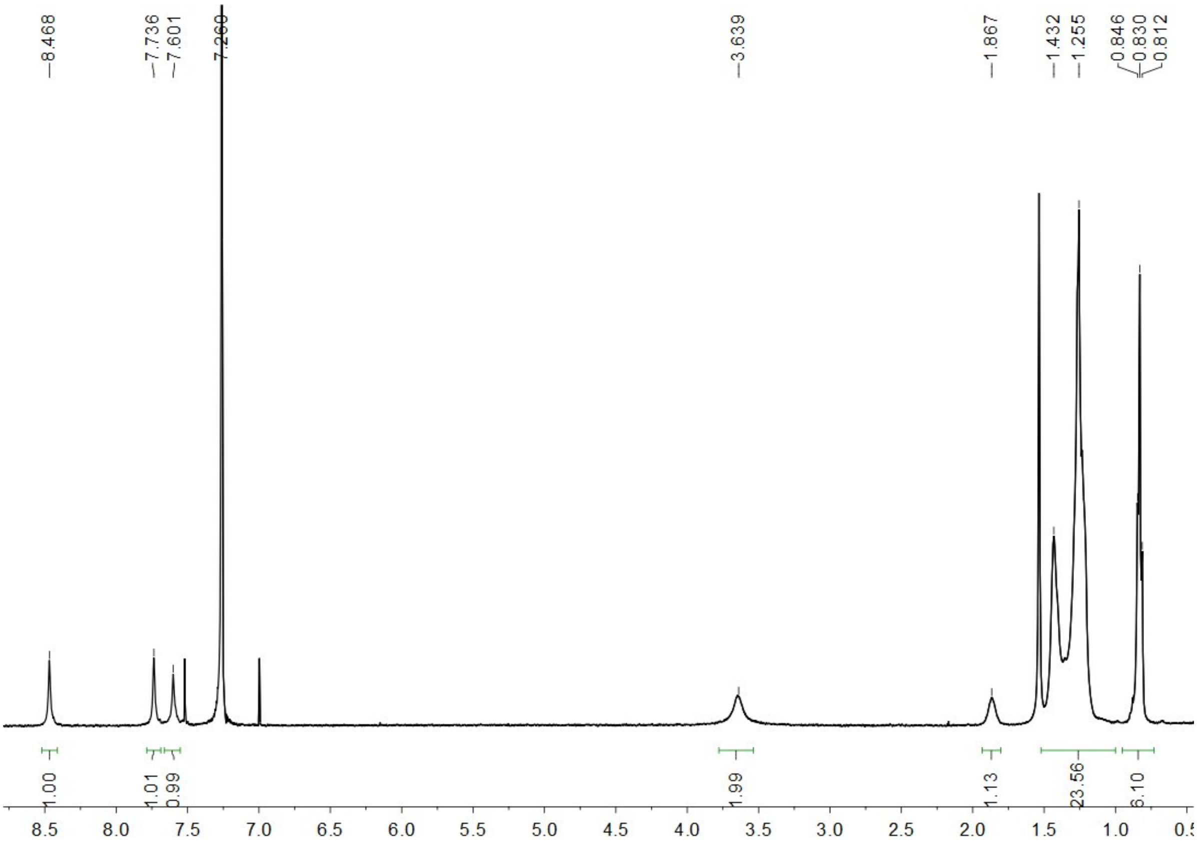

Figure S10. ^{1}H NMR spectrum of **P-BNBP-T**.
